# High-performance silicon polarization switch based on a Mach–Zehnder interferometer integrated with polarization-dependent mode converters

**DOI:** 10.1515/nanoph-2022-0022

**Published:** 2022-03-11

**Authors:** Weike Zhao, Ruoran Liu, Yingying Peng, Xiaolin Yi, Haitao Chen, Daoxin Dai

**Affiliations:** State Key Laboratory for Modern Optical Instrumentation, Center for Optical & Electromagnetic Research, College of Optical Science and Engineering, International Research Center for Advanced Photonics, Zhejiang University, Zijingang Campus, Hangzhou 310058, China; Ningbo Research Institute, Zhejiang University, Ningbo 315100, China

**Keywords:** Mach–Zehnder interferometer, mode hybridness, polarization, silicon, switch

## Abstract

As the key element for optical systems, polarization controllers with versatile functionalities are highly desired. Here, a CMOS-compatible polarization switch is proposed and realized by using a Mach–Zehnder interferometer integrated with two polarization-dependent mode converters (PDMCs) at the input/output ends. The PDMCs, which utilize the mode hybridness and adiabatic mode evolution in a silicon-on-insulator (SOI) ridge waveguide taper, provide a low-loss adiabatic transmission for the launched TE_0_ mode as well as efficient mode conversion from the launched TM_0_ mode to the TE_1_ mode. For the MZI structure, there are two 1 × 2 dual-mode 3-dB power splitters based on a triple-core adiabatic taper, and two thermally-tunable phase-shifters embedded in the arms. The polarization state and the polarization extinction ratio (PER) of the transmitted light can be dynamically tuned by introducing some phase difference between the MZI arms electrically. The fabricated device has an excess loss of ∼0.6 dB for the TE_0_ and TM_0_ modes. When the switch is off, the TE_0_ and TM_0_ modes go through the device without exchange. In contrast, when the switch is on, the TE_0_–TM_0_ conversion occurs and the measured PER is about 20 dB.

## 1 Introduction

Silicon photonics have attracted more and more attention because it is emerging as one of the most prospective optoelectronic integrated platforms due to its high integration density and CMOS compatibility [1], [2], [3]. However, silicon photonic devices often severely suffer from polarization mode dispersion and polarization-dependent loss due to the ultra-high index-contrast and structural asymmetry of silicon photonic waveguides [4]. A general solution for this issue is using a polarization diversity system that contains polarization control devices like polarizer [5], [6], [7], polarization beam splitters (PBSs) [8], [9], [10], polarization rotators (PRs) [11, 12], as well as polarization splitter-rotators (PSRs) [13], [14], [15], etc. To date, PRs have been demonstrated by using vertical asymmetric structures, including double-core waveguides [11], metasurface waveguides [12], adiabatic tapers [13], as well as asymmetric directional couplers [14]. However, most of these reported PRs are static, while reconfigurable polarization switches, which can dynamically tune the ratio of two polarization components, are highly sought in various applications including polarization-diversity optical networks [16], [17], [18], polarization-encoded quantum technologies [19], [20], [21], [22], [23], and polarization-switched coherent communications [24], [25], [26], [27], etc. Until now, there have been only a very few polarization switches reported [28], [29], [30], [31], [32]. In Ref. [28], a polarization switch based on mode hybridness was proposed by incorporating two polarization rotator-splitters and two MZIs. It has a polarization extinction ratio (PER) of 13 dB and an excess loss (EL) of 2.5 dB. In Ref. [29], an electrically-tunable polarization switch was demonstrated by introducing an out-of-plane optical waveguide to access berry’s phase. It has a PER of 10 dB and an EL of ∼1 dB. In Ref. [30], a polarization switch with a PER of ∼20 dB was demonstrated by cascading three PRs based on a partially-etched waveguide and three electrically-tunable phase-shifters, which however is complex and as long as thousands of micrometers. Therefore, a compact polarization switch with high performances such as high PERs, low ELs and broad bandwidths is still absent.

In this work, we propose and demonstrate a compact and CMOS-compatible silicon polarization switch that can be reconfigured thermally. The proposed device is constructed with a Mach–Zehnder interferometer (MZI) based on bi-level ridge waveguides and two polarization-dependent mode converters (PDMCs) at the input/output ends. The PDMCs enable the mode conversion between the TM_0_ and TE_1_ modes, and also provide an adiabatic transmission for the launched TE_0_ mode. For the MZI structure, there are two 1 × 2 dual-mode 3-dB power splitters (PSs) based on a triple-core adiabatic taper, and two thermally-tunable phase-shifters embedded in the arms. In particular, the dual-mode 3-dB couplers split the TE_0_ or TE_1_ modes into two TE_0_ modes equally. The phase-shifters are used to control the phase difference of these two TE_0_ modes propagating along the MZI arms. In this way, the polarization ratio of light can be effectively manipulated as desired. The device fabricated with CMOS-compatible foundry processes shows an EL of ∼1.2 dB for the TE_0_ and TM_0_ modes and the power consumption for switching is about 26 mW. The PER of the present polarization switch is about 20 dB in the wavelength range of 1530–1600 nm for both polarizations at the on/off states.

## 2 Principle and structural design

**Figure 1:**
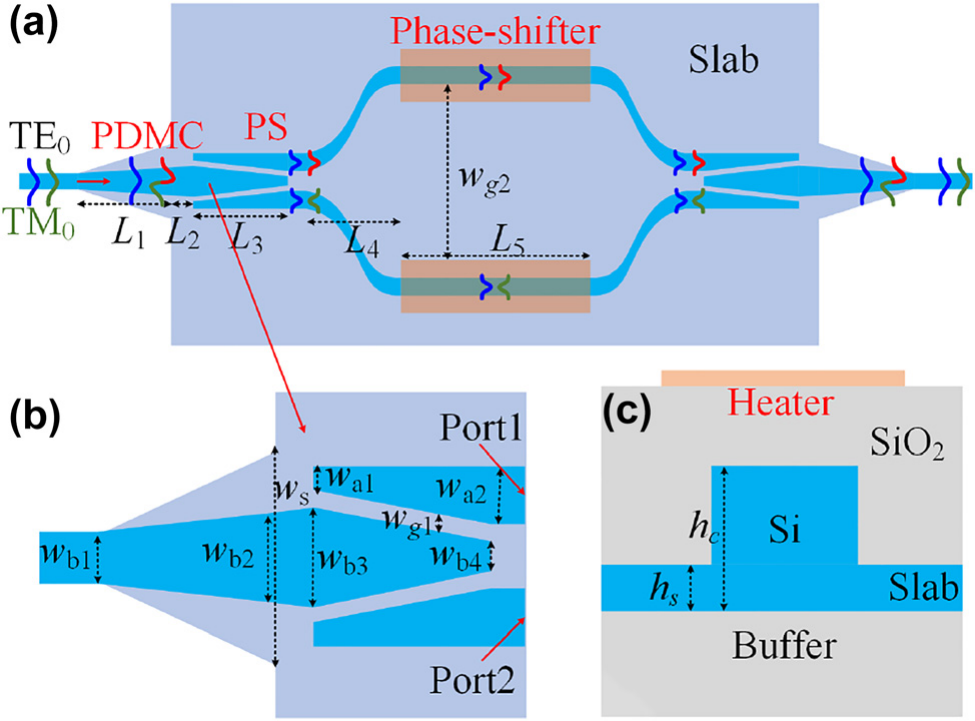
Schematic configuration of the proposed polarization switch. (a) The top view; (b) the PDMC and PS regions; and (c) the cross-section of the ridge waveguide of the phase shifter.

Figure 1a shows the top view of the proposed polarization switch, which consists of a MZI connected with two PDMCs at the input/output ends. The MZI is constructed with two 1 × 2 dual-mode 3-dB PSs based on a triple-core adiabatic taper and two thermally-tunable phase-shifters embedded in the arms. Figure 1b and c show the enlarged views of the PDMC and PS as well as the cross-section of the phase-shifter. Here, we use SOI ridge waveguides, which have a core height of *h*_c_ = 220 nm and a slab height of *h*_s_ = 70 nm, making it compatible with the standard foundry process. The buried oxide layer and the silica upper cladding layer are both 2 μm, and the metal micro-heater of the phase-shifter is located upon the silica upper-cladding of the MZI’s arms.

As shown in Figure 1a, when the TE_0_ mode is launched, it passes through the PDMC adiabatically and is split into two parts with the same phase by the triple-core PS. These two parts of power are carried by the TE_0_ modes and then recombined to the TE_0_ or TE_1_ modes, which depends on the phase-shifting Δ*φ* introduced thermally by the phase-shifter. When Δ*φ* = 0, one has the TE_0_ mode at the output of the 2 × 1 triple-core PS and finally outputs as the TE_0_ mode even with the PDMC. When Δ*φ* = *π*, one has the TE_1_ mode at the output of the 2 × 1 triple-core PS and finally converted to be the TM_0_ mode with the PDMC at the output end. In contrast, the launched TM_0_ mode is converted to the TE_1_ mode by the PDMC, and then is split into two parts with a phase difference of *π* by the triple-core PS. These two parts of power are carried by the TE_0_ modes and then recombined to the TE_0_ or TE_1_ mode, depending on the phase-shifting Δ*φ*. When Δ*φ* = 0, one has the TE_1_ mode at the output of the 2 × 1 triple-core PS and finally converted to be the TM_0_ mode with the PDMC at the output end. When Δ*φ* = *π*, one has the TE_0_ mode at the output of the 2 × 1 triple-core PS and finally outputs as the TE_0_ mode even with the PDMC. In this way, we can selectively realize the polarization conversion of the TE_0_–TM_0_ modes by thermally controlling the phase difference Δ*φ* of the two arms of the MZI.

**Figure 2:**
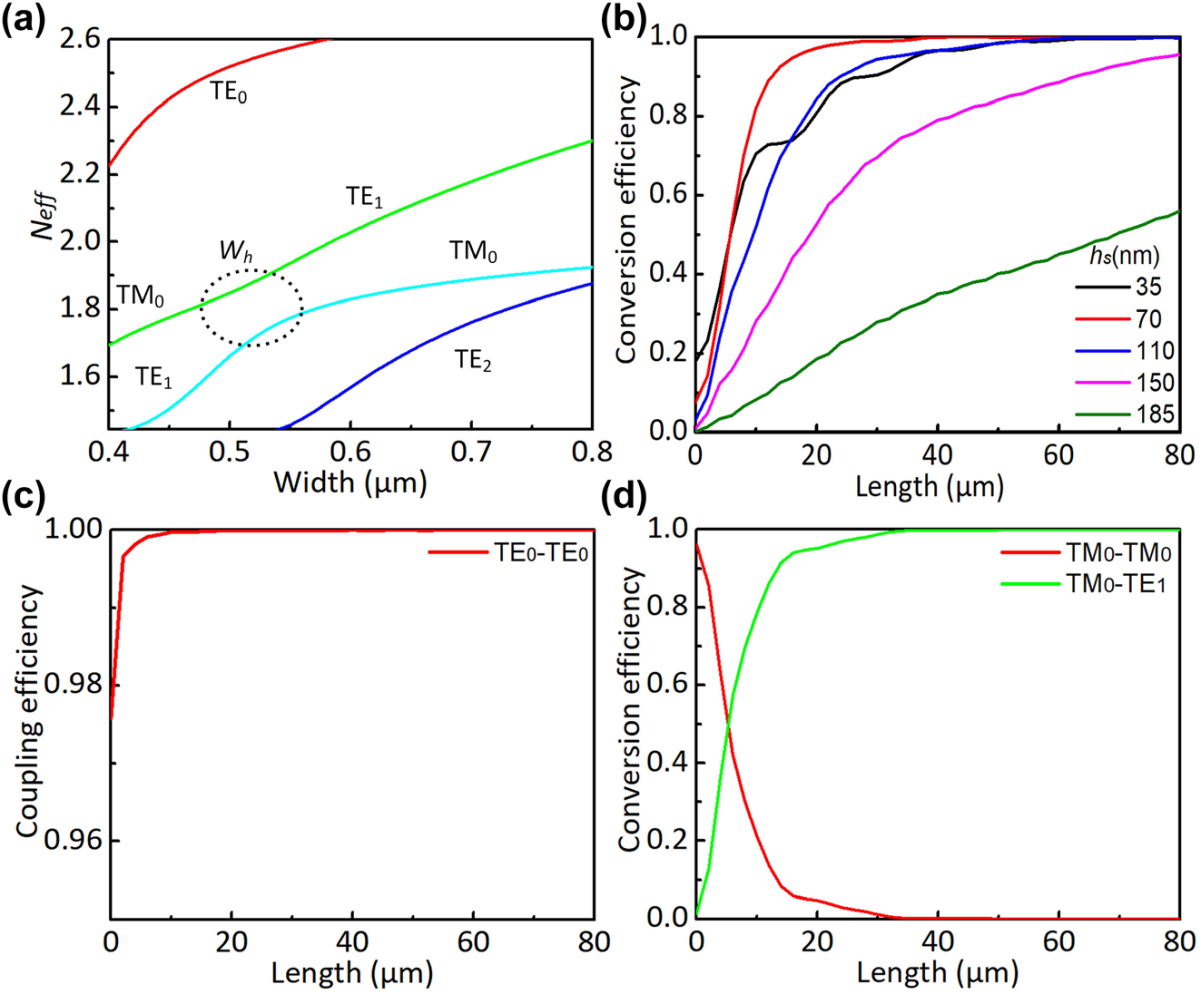
Design of the PDMC. (a) Calculated mode effective index *n*_eff_ for the bi-level ridge waveguide; (b) calculated TM_0_–TE_1_ mode conversion efficiency as the taper length *L*_1_ varies for different slab heights *h*_s_; (c) calculated TE_0_–TE_0_ mode conversion efficiency as the taper length *L*_1_ varies (here, *h*_s_ = 70 nm); and (d) calculated TM_0_–TE_1_ mode conversion efficiency as the taper length *L*_1_ varies (here, *h*_s_ = 70 nm).

**Figure 3:**
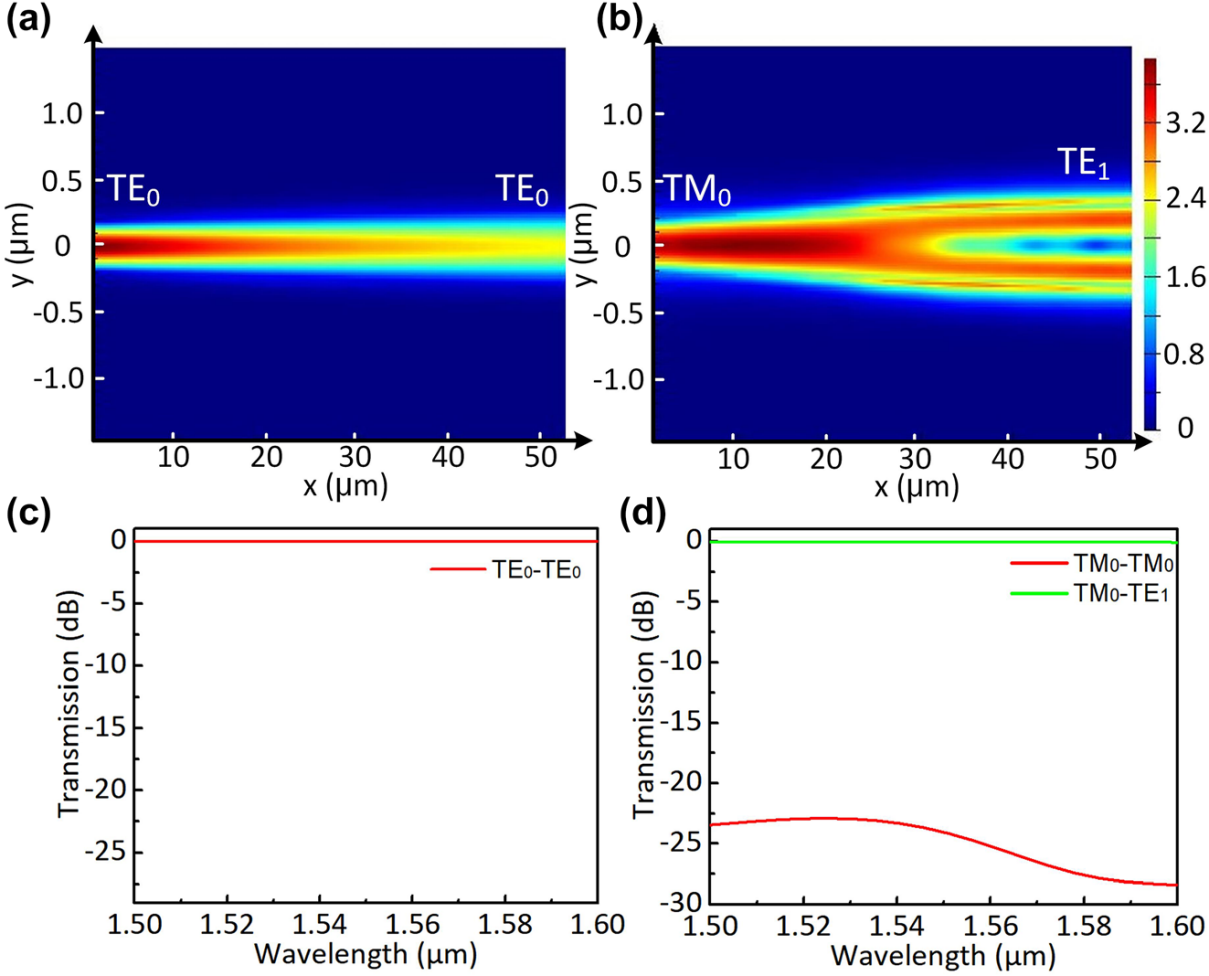
Simulated light propagation in the designed PDMC for the launched TE_0_ (a) and TM_0_ (b) modes with the FDTD method; and calculated transmission spectra in the designed PDMC for the launched TE_0_ (c) and TM_0_ (d) modes.

The PDMC design is based on the mode hybridness and the mode evolution in an adiabatic taper based on an SOI ridge waveguide with vertical asymmetry, as proposed previously [28]. Here, a bi-level tapered waveguide with a length of *L*_1_ is used, as shown in Figure 1b. The width of the top-ridge is linearly tapered from *w*_b1_ to *w*_b2_ while the bottom-ridge is accordingly tapered from *w*_b1_ to *w*_s_. The calculated mode effective index *n*_eff_ for the bi-level ridge waveguide is shown in Figure 2a. It can be seen that the dispersion curves for the TM_0_ and TE_1_ modes are close to each other when the waveguide width is around *w*_h_ = 0.52 μm, where the mode hybridness happens. In order to achieve the TM_0_–TE_1_ mode conversion, we choose the taper end-widths as *w*_b1_ = 0.4 μm and *w*_b2_ = 0.8 μm, so that the taper width *w*_h_ is in the range of *w*_b1_ < *w*_h_ < *w*_b2_. Meanwhile, the bottom-ridge width *w*_s_ at the taper end is chosen as wide as 2 μm. The TM_0_–TE_1_ mode conversion efficiency is calculated with the eigenmode expansion (EME) solver for the case with different slab heights of *h*_s_ = 35, 70, 110, 150 nm as the length *L*_1_ varies, as shown in Figure 2b. It can be seen that the length for the PDMC can be minimized when choosing *h*_s_ = 70 nm. Furthermore, the height of *h*_s_ = 70 nm is advisable according to the standard MPW process provided by the foundry. As a result, we choose the slab height as *h*_s_ = 70 nm. Then the calculated TE_0_–TE_0_ and TM_0_–TE_1_ mode conversion efficiencies are shown in Figure 2c and d. It can be seen that the efficiency for the TE_0_–TE_0_ mode conversion is higher than 99.9% when *L*_1_ > 20 μm, as shown in Figure 2b. On the other hand, the efficiency for the TM_0_–TE_1_ mode conversion is larger than 99.9% when *L*_1_ > 50 μm, as shown in Figure 2c. Therefore, we choose *L*_1_ = 60 μm to have high efficiency for both TE_0_–TE_0_ and TM_0_–TE_1_ mode conversions as well as large fabrication tolerances for the core-width deviations. Light propagation in the designed PDMC is simulated for the launched TE_0_ and TM_0_ modes with the FDTD method, as shown in Figure 3a and b. The corresponding transmission spectra are shown in Figure 3c and d. It can be seen that the launched TE_0_-mode transmission is lossless almost while the launched TM_0_ mode is converted to the TE_1_ mode with an ultra-low loss less than 0.08 dB and a high extinction ratio (ER) larger than 22 dB in a broad wavelength range of 1500–1600 nm.

In order to simultaneously split the TE_0_ and TE_1_ modes with a splitting ratio of 50%:50%, the PS should be designed carefully. However, the design of using a regular directional coupler, a regular multimode interferometer as well as a regular Y-branch does not work well for both modes due to the high loss. Here, a 1 × 2 PS based on a triple-core adiabatic taper is introduced [33], as shown in Figure 1b. The middle core is tapered linearly from *w*_b3_ to *w*_b4_ with a length of *L*_3_, while the two identical side cores are from *w*_a1_ to *w*_a2_. The gap between the middle core and the side core has a uniform width of *w*_g1_. In the triple-core taper, the TE_0_ or TE_1_ modes launched from the middle core are gradually converted to two decoupled TE_0_ modes supported in the two side cores. These two decoupled TE_0_ modes have a phase difference of 0 or π. The PS can be designed according to the following guidance.

(1) The width *w*_b3_ at the input end should be wide enough and the width *w*_a1_ should be narrow enough so that the two lowest-order supermodes at the input end are localized very well in the middle core. In this way, the mode mismatch at the junction connecting to the input section can be minimized.
(2) The width *w*_b4_ at the output end should be narrow enough and the width *w*_a2_ should be wide enough so that the two lowest-order supermodes are localized very well in the side cores. In this way, the mode mismatch at the junction connecting to the output section can be minimized.
(3) The length *L*_3_ should be long enough to ensure the adiabatic transition for the TE_0_ and TE_1_ modes.

**Figure 4:**
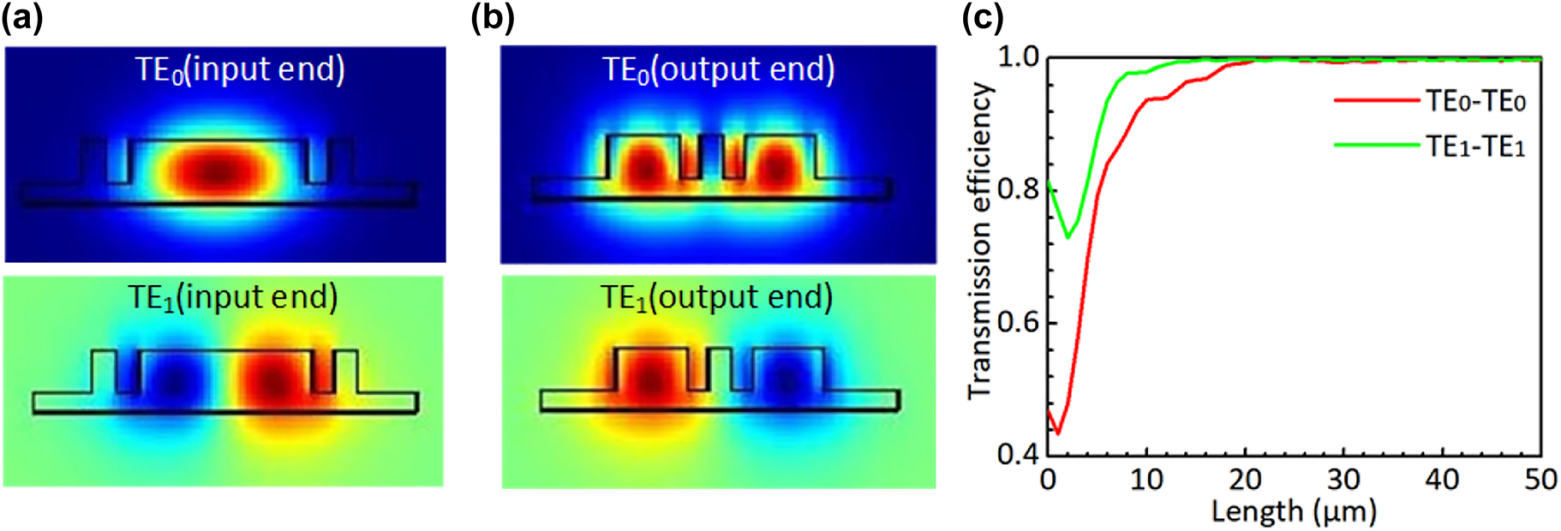
The mode profiles of the TE_0_ and TE_1_ modes at the input end (a) and the output end (b) of the triple-core waveguide of the PS. (c) Simulated conversion efficiencies of the TE_0_–TE_0_ and TE_1_–TE_1_ in the triple-core waveguide as the length *L*_3_ varies.

**Figure 5:**
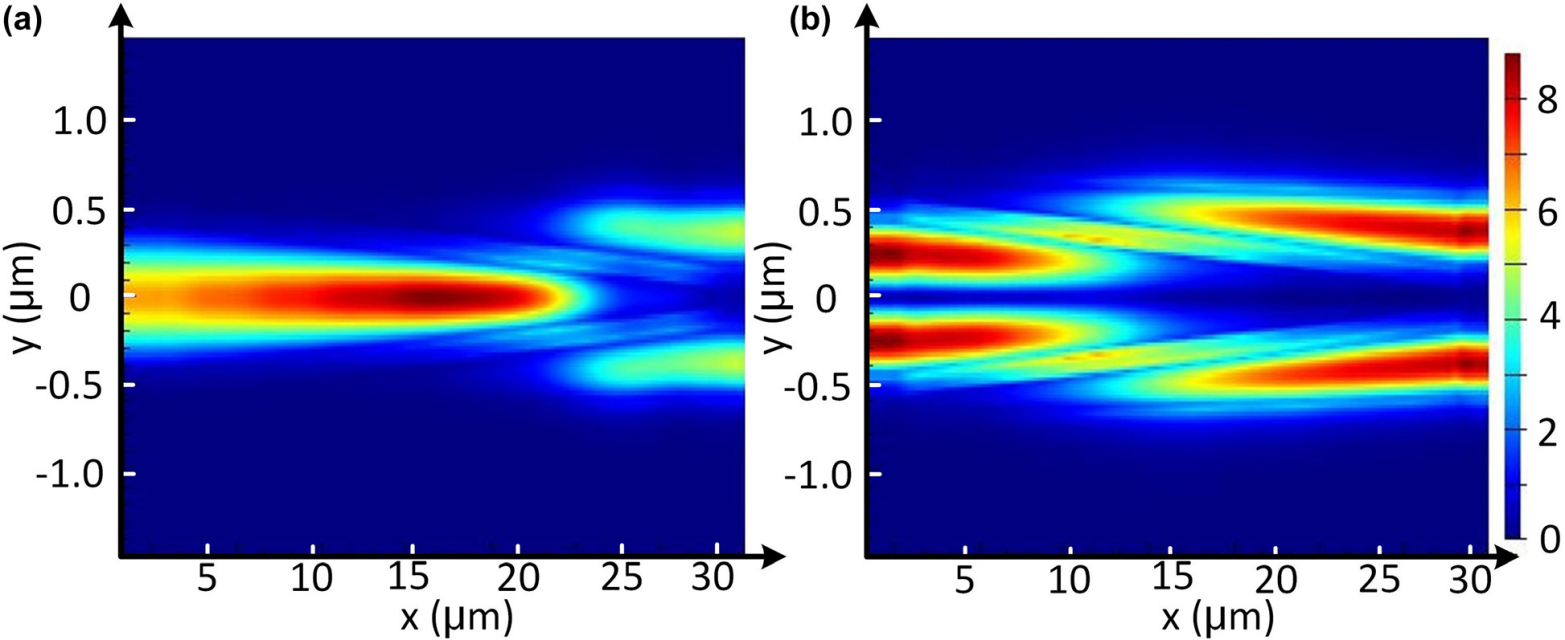
Simulated light propagation in the designed power splitter for the launched TE_0_ (a) and TE_1_ (b) modes.

**Figure 6:**
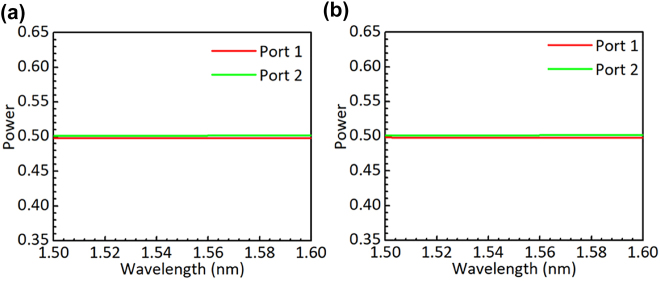
Simulated power splitting ratio of the designed power splitter for the TE_0_ (a) and TE_1_ (b) modes.

Considering the fabrication limitation, the widths *w*_b4_, *w*_a1_ and *w*_g1_ are chosen to be 120 nm. Our calculation shows that >99% power of the TE_0_ and TE_1_ modes are well confined in the middle core region when *w*_b3_ = 0.9 μm at the input end. Similarly, we choose *w*_a2_ = 0.4 μm at the output end. Figure 4a and b shows the calculated mode profiles of the TE_0_ and TE_1_ modes at the input and output ends of the triple-core PS. As expected, these modes at the input end are well confined in the middle core region [see Figure 4a] and these modes at the output end are well confined in the side-core region [see Figure 4b]. Therefore, the mode-mismatch loss at the junctions connecting to the input/output sections is very low. Figure 4c shows the transmissions in the PS as the length *L*_3_ varies. The transmission efficiencies of the TE_0_ and TE_1_ modes are both higher than 99% when *L*_3_ > 25 μm. Figure 5a and b show the simulated light propagations of the TE_0_ and TE_1_ modes launched from the input end of the designed PS with *L*_3_ = 30 μm by using the FDTD method. The calculated power splitting ratios for the TE_0_ and TE_1_ modes are shown in Figure 6a and b. As it can be seen, the designed PS can split the TE_0_ and TE_1_ modes equally with a very low EL less than 0.01 dB in the wavelength range of 1500–1600 nm.

**Figure 7:**
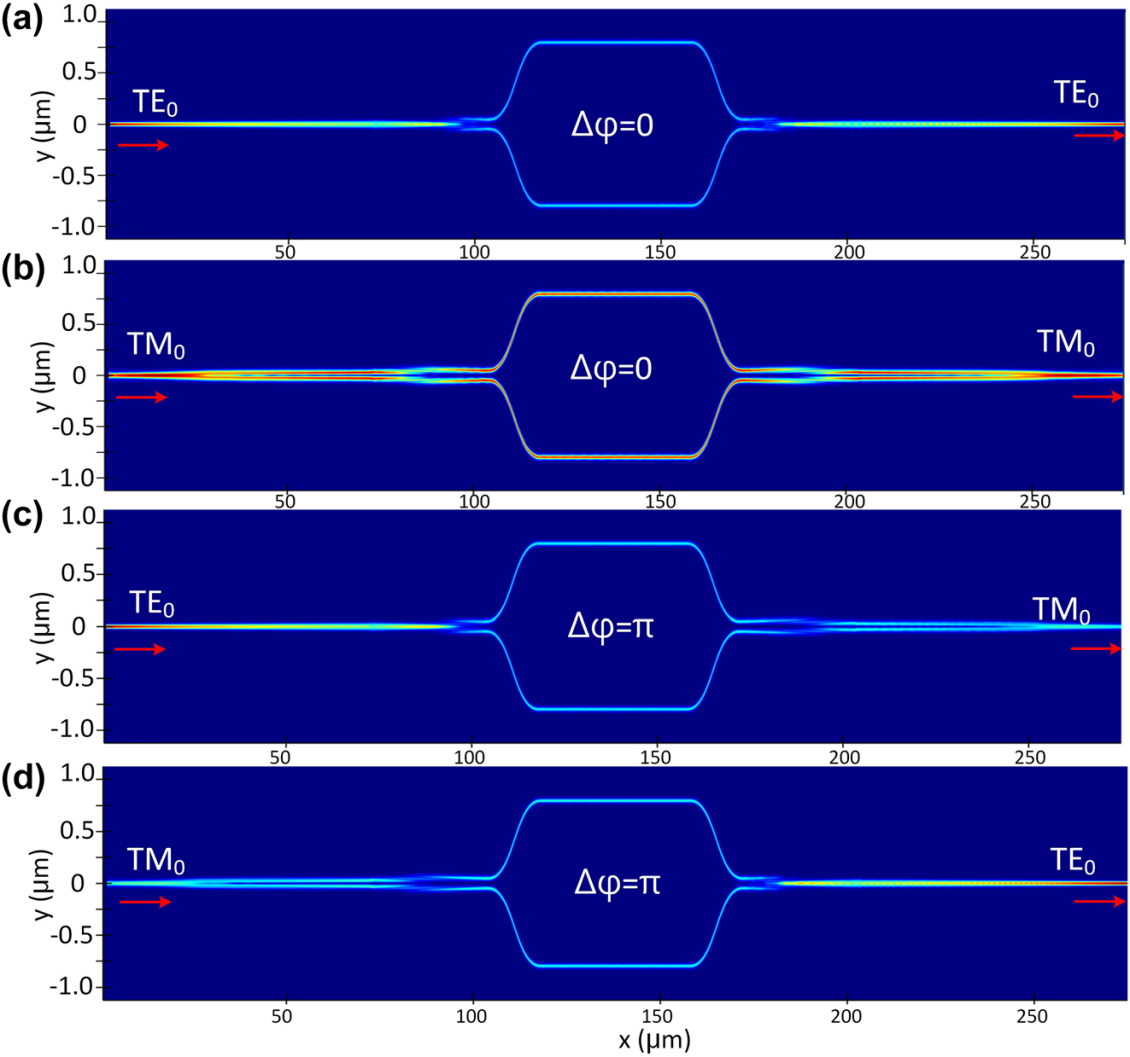
Simulated light propagation of the designed polarization switch for the launched TE_0_ (a) and TM_0_ (b) modes with Δ*φ* = 0; simulated light propagation of the designed polarization switch for the launched TE_0_ (c) and TM0 (d) modes with Δ*φ* = *π*.

**Figure 8:**
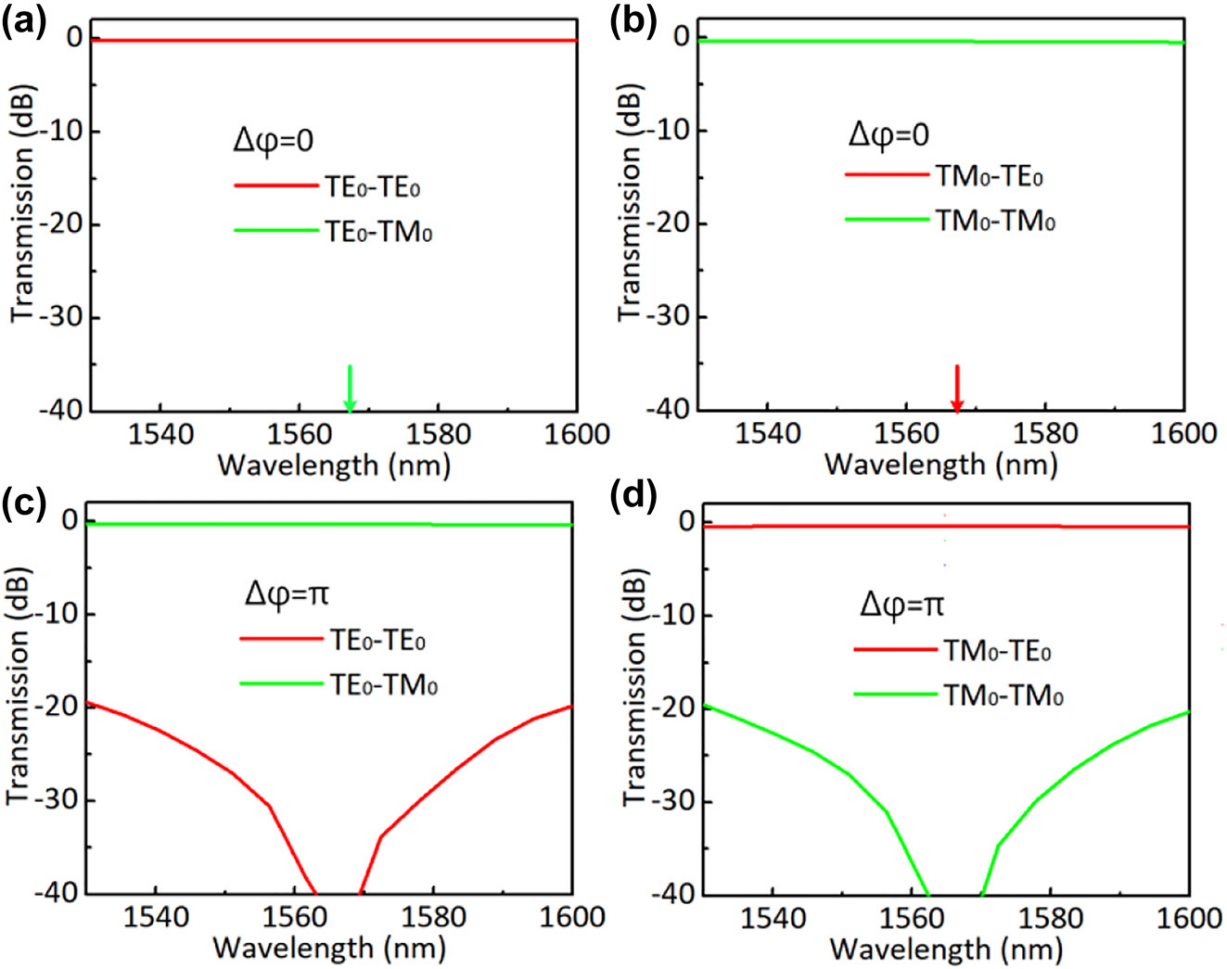
Simulated light transmissions of the designed polarization switch for the launched TE_0_ (a) and TM_0_ (b) modes with Δ*φ* = 0; simulated light propagation of the designed polarization switch for the launched TE_0_ (c) and TM_0_ (d) modes with Δ*φ* = *π*.

The phase-shifters of the MZI works based on the thermo-optic effect of silicon photonic waveguides. One arm of the MZI is heated by the metal micro-heater to introduce the desired phase difference Δ*φ* of 0 or *π*, depending on the injecting current*.* The gap *w*_g2_ between the two arms is chosen as 12 μm to decrease the thermal crosstalk, while the length of the S bends is 14 μm to guarantee adiabatic transition. The length of the two arms is chosen as 40 μm, and the total length for the designed polarization switch is about 246 μm. Table 1 gives all the key parameters for the designed polarization switch. When there is no phase difference introduced in the phase-shifter, the propagation of the launched TE_0_ and TM_0_ modes in the proposed polarization switch are shown in Figure 7a and b, respectively. Here, the input TE_0_ and TM_0_ modes finally output from the output port without polarization conversion. Figure 7c and d show the simulated light propagation of the launched TE_0_ and TM_0_ modes in the designed polarization switch when a phase difference of *π* is introduced between the MZI arms. Here, it can be seen the launched TE_0_ and TM_0_ modes are converted successfully. Figure 8a and b show the corresponding transmissions for the launched TE_0_ and TM_0_ modes when Δ*φ* = 0. In this case, both TE_0_ and TM_0_ modes have a low EL of <0.45 dB (>90%) and a very high PER of >90 dB in an ultra-broad wavelength range. Figure 8c and d show the corresponding transmissions for the launched TE_0_ and TM_0_ modes when Δ*φ* = *π*. In this case, the TE_0_–TM_0_ and TM_0_–TE_0_ mode conversions have a low EL of <0.4 dB (>91%) and a high PER of >20 dB in the wavelength range of 1530–1600 nm. The bandwidth is mainly limited by the dispersion of MZI structure and can be extended by further shorting the MZI’s arm length *L*_5_. The fabrication tolerance of the proposed polarization switch is mainly determined by the PDMCs and the PSs. These two parts both work with the principle of adiabatic mode evolution, which has been proved to be fabrication-tolerant [28, 33]. Therefore, the proposed polarization switch has a large fabrication tolerance in principle.

**Table 1:** Key parameters of the designed polarization switch.

Parameters	*w*_b1_	*w*_b2_	*w*_b3_	*w*_b4_	*w*_S_	*w*_a1_	*w*_a2_	*w*_g1_
Values (μm)	0.4	0.8	0.9	0.12	2	0.12	0.14	0.12

Parameters	*w*_g2_	*L*_1_	*L*_2_	*L*_3_	*L*_4_	*L*_5_
Values (μm)	12	60	1	30	12	40

## 3 Fabrication and results

**Figure 9:**
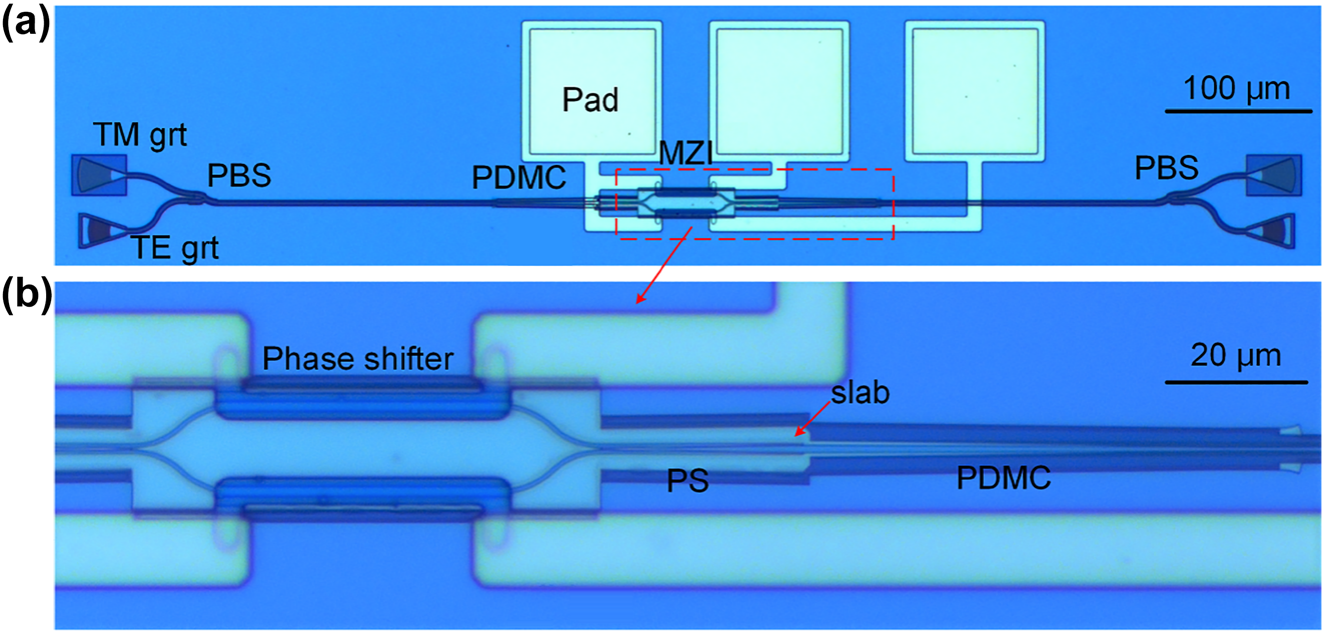
The optical microscopy images of the fabricated polarization switch. (a) The optical microscopy images for the fabricated polarization switch with two PBSs; and (b) the enlarged view of MZI with power splitters and phase shifters.

The designed device was then fabricated with the E-beam lithography foundry process. Metal micro-heaters of Cr (20 nm)/Ti (200 nm) alloy are used as a heater. Figure 9a shows the optical microscopy images of the fabricated device. Two high-performance PBSs [9] with efficient TE-/TM-type grating couplers are connected at the input/output ports of the present device to conveniently characterize the responses of the TE_0_/TM_0_ modes. Figure 9b shows the enlarged view of the MZI consisting of the dual-mode 3-dB PSs and the phase shifters.

**Figure 10:**
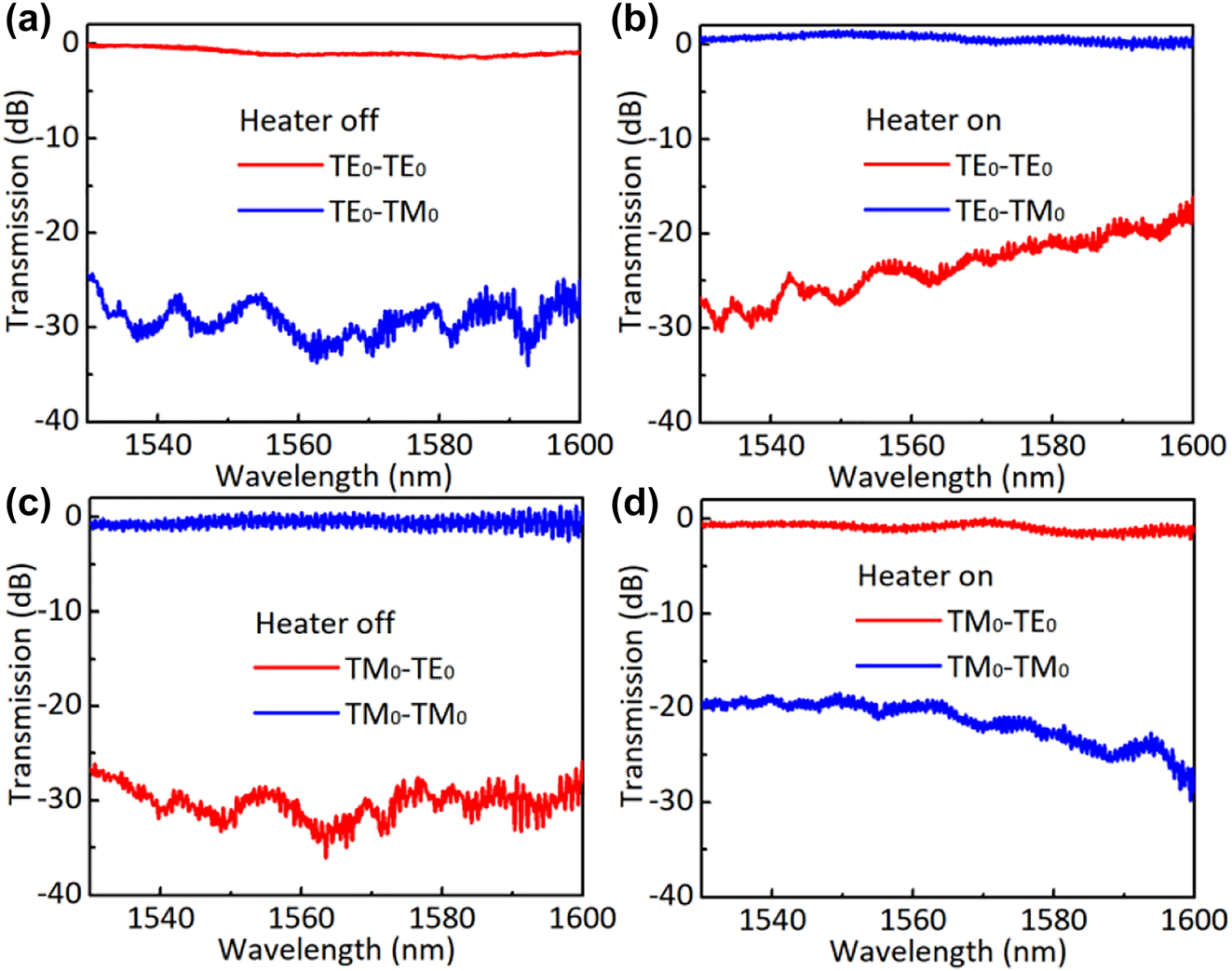
Measured transmissions of the fabricated polarization switch for the launched TE_0_ (a) and TM_0_ (b) modes when it is off; measured transmissions of the fabricated polarization switch for the launched TE_0_ (c) and TM_0_ (d) modes when it is on.

For the characterization of the fabricated devices, an amplified spontaneous emission (ASE) light source was used. The polarization state of light is adjusted to the desired one by using a fiber polarizer and a polarization controller. The polarized light is then coupled to the chip through the TE- or TM-type gratings. At the output side, light is routed to the port corresponding to the TE_0_ or TM_0_ modes by using a PBS and analyzed by an optical spectrum analyzer. The measured transmissions are normalized by a straight waveguide fabricated on the same chip. Figure 10a shows the measured transmissions at the output ports for the launched TE_0_ mode when the heater is off. In this case, no polarization rotation is observed almost, and the switch has a low loss of ∼1.2 dB and a PER higher than 25 dB in the wavelength range of 1530–1600 nm. By excluding the EL of ∼0.6 dB from the two PBSs, the present polarization switch itself has a low EL of ∼0.6 dB. The low EL is attributed that the PRs and the PSs working with the adiabatic mode evolution principle and respectively have ultra-low Els of <0.08 dB and <0.01 dB in theory. When the heater is on with a power 26 mW, the launched TE_0_ mode is switched to the TM_0_ mode with a PER of >20 dB, as shown in Figure 10b.

Figure 10c and d show the measured transmission at the output ports for the launched TM_0_ mode when the heater is off and on, respectively. When the heater is off, one has the TM_0_ mode at the output port with a low loss of ∼1.1 dB and a high PER of >28 dB. As shown in Figure 10d, when the heater is on, one has an efficient TM_0_–TE_0_ mode conversion with a PER of >19 dB. When the heating power is varied from 0 to 26 mW, the PER of light at the output port can be tuned freely and a tunable PR is achieved. The measured transmissions do not show a deep notch in the wavelength around the desired central wavelength as shown in Figure 10b and d, because the power splitting ratio is not 50%:50% perfectly due to the fabrication error and a perfect phase-shifting of *π* is not achieved at the desired wavelength due to the inaccurate calibration in the experiment. Table 2 gives a summary of those reported polarization switches. As shown in Table 2, the present device shows the best overall performance, like compact footprints, high PERs, and low losses. Especially, it has the largest bandwidth, owing to the introduction of an adiabatic 3-dB triple-core PS.

**Table 2:** Summary of polarization switches on silicon.

Operating principle	PER (dB)	Length (μm)	Bandwidth (nm)	EL (dB)
Mode hybridness [28]	13	∼3000	@1570	2.5
Berry’s phase [29]	10	150	@1556	1
Partial etching and phase shifter [30]	20	3000	@1550	0.7
This work	20	246	>70 nm	∼0.6

## 4 Conclusions

In conclusion, we have proposed and demonstrated a novel high-performance polarization switch by using a 1 × 1 MZI integrated with two PDMCs at the input/output ends. The PDMCs have been designed to enable a low-loss adiabatic transmission for the launched TE_0_ mode and an efficient mode conversion from the launched TM_0_ mode to the TE_1_ mode by utilizing the mode hybridness and the adiabatic mode evolution in an SOI ridge waveguide taper. For the present MZI, two 1×2 dual-mode 3-dB PSs have been introduced with low ELs and uniform power splitting for the TE_0_ and TE_1_ modes by using a triple-core adiabatic taper. It has been demonstrated that the polarization states of light can be dynamically switched by tuning the phase difference between the MZI arms. The ELs are about 0.6 dB and the PERs are >20 dB for both TE_0_ and TM_0_ modes in the wavelength range of 1530–1600 nm. Furthermore, the present polarization switch has a compact footprint of 246 × 150 μm^2^. It will be useful as a key element in many on-chip photonic systems.
